# Endosymbiont Capture, a Repeated Process of Endosymbiont Transfer with Replacement in Trypanosomatids *Angomonas* spp.

**DOI:** 10.3390/pathogens10060702

**Published:** 2021-06-04

**Authors:** Tomáš Skalický, João M. P. Alves, Anderson C. Morais, Jana Režnarová, Anzhelika Butenko, Julius Lukeš, Myrna G. Serrano, Gregory A. Buck, Marta M. G. Teixeira, Erney P. Camargo, Mandy Sanders, James A. Cotton, Vyacheslav Yurchenko, Alexei Y. Kostygov

**Affiliations:** 1 Institute of Parasitology, Biology Centre, Czech Academy of Sciences, 370 05 České Budějovice (Budweis), Czech Republic; Tomas.Skalicky@seznam.cz (T.S.); rolando24@yandex.ru (A.B.); jula@paru.cas.cz (J.L.); 2 Department of Parasitology, Institute of Biomedical Sciences, University of São Paulo, São Paulo 05508-000, Brazil; jotajj@usp.br (J.M.P.A.); acm2911@usp.br (A.C.M.); mmgteix@icb.usp.br (M.M.G.T.); erney@usp.br (E.P.C.); 3 Life Science Research Centre, Faculty of Science, University of Ostrava, 710 00 Ostrava, Czech Republic; janna.krallova@gmail.com (J.R.); vyacheslav.yurchenko@osu.cz (V.Y.); 4 Faculty of Sciences, University of South Bohemia, 370 05 České Budějovice (Budweis), Czech Republic; 5 Department of Microbiology and Immunology, Virginia Commonwealth University, Richmond, VA 23298-0678, USA; myrna.serrano@vcuhealth.org (M.G.S.); gregory.buck@vcuhealth.org (G.A.B.); 6 Wellcome Sanger Institute, Wellcome Genome Campus, Hinxton, Cambridge CB10 1SA, UK; mjs@sanger.ac.uk (M.S.); james.cotton@sanger.ac.uk (J.A.C.); 7 Martsinovsky Institute of Medical Parasitology, Sechenov University, 119435 Moscow, Russia; 8 Zoological Institute of the Russian Academy of Sciences, 199034 St. Petersburg, Russia

**Keywords:** genome, bacterial endosymbionts, Trypanosomatidae, *Angomonas*

## Abstract

Trypanosomatids of the subfamily Strigomonadinae bear permanent intracellular bacterial symbionts acquired by the common ancestor of these flagellates. However, the cospeciation pattern inherent to such relationships was revealed to be broken upon the description of *Angomonas ambiguus*, which is sister to *A. desouzai*, but bears an endosymbiont genetically close to that of *A. deanei*. Based on phylogenetic inferences, it was proposed that the bacterium from *A. deanei* had been horizontally transferred to *A. ambiguus*. Here, we sequenced the bacterial genomes from two *A. ambiguus* isolates, including a new one from Papua New Guinea, and compared them with the published genome of the *A. deanei* endosymbiont, revealing differences below the interspecific level. Our phylogenetic analyses confirmed that the endosymbionts of *A. ambiguus* were obtained from *A. deanei* and, in addition, demonstrated that this occurred more than once. We propose that coinfection of the same blowfly host and the phylogenetic relatedness of the trypanosomatids facilitate such transitions, whereas the drastic difference in the occurrence of the two trypanosomatid species determines the observed direction of this process. This phenomenon is analogous to organelle (mitochondrion/plastid) capture described in multicellular organisms and, thereafter, we name it endosymbiont capture.

## 1. Introduction

The flagellates of the family Trypanosomatidae are well known for human pathogens, such as *Trypanosoma brucei*, *T. cruzi*, and various *Leishmania* spp., yet the majority of trypanosomatid genera are intestinal parasites of insects [1]. In the process of adaptation to this omnipresent and extremely diverse group of hosts, trypanosomatids acquired many peculiar features, the study of which illuminated not only the evolution of parasitism in this group, but also the evolutionary strategies of eukaryotes in general [2,3]. One of the most intriguing phenomena is the presence of bacteria in the cytoplasm of some of these flagellates [4]. Such symbiotic relationships originated in trypanosomatids several times independently and range from recently established and unstable ones to those that demonstrate a high level of integration [5,6,7,8]. Mutualistic nature of these endosymbioses is demonstrated by the metabolic cooperation between the bacteria and their trypanosomatid hosts, removing the dependence of the latter on the environmental availability of essential nutrients, such as heme, some amino acids, and vitamins [9,10,11,12].

The first discovered and, consequently, most studied group of endosymbiont-bearing trypanosomatids is the subfamily Strigomonadinae, comprising seven described species of the genera *Angomonas*, *Strigomonas,* and *Kentomonas* [7,13]. All of these species have intracytoplasmic bacteria *Candidatus* Kinetoplastibacterium spp. belonging to the family Alcaligenaceae (Betaproteobacteria: Burkholderiales), and, as judged by their respective phylogenies, the origin of the endosymbiosis was a single event followed by a prolonged coevolution [14]. However, the description of *Angomonas ambiguus* revealed a violation of the co-speciation pattern: being a sister species to *A. desouzai*, this flagellate contained an endosymbiont not discernible from that of *Angomonas deanei* by the sequences of the 16S ribosomal RNA gene and the internal transcribed spacer [7]. This discrepancy was reflected in the name of the described trypanosomatid (meaning “ambiguous” in Latin). The endosymbionts of both *A. deanei* and *A. ambiguus* were classified into a single species, *Ca*. Kinetoplastibacterium crithidii [7]. When the same discordance was later shown in the phylogenies of trypanosomatids and their endosymbionts based on the glyceraldehyde 3-phosphate dehydrogenase (GAPDH) gene, it was proposed that *A. ambiguus* obtained its endosymbiont from *A. deanei* by horizontal transfer [15].

In this work, we address these complex evolutionary relationships by analyzing the genomic sequences of two strains of *A. ambiguus* and their respective endosymbionts from geographically distant locations (Brazil and Papua New Guinea) using comparative genomic and phylogenetic tools. Our results not only confirm the transition of bacteria between the two *Angomonas* species, but also demonstrate that this was not a singular event.

## 2. Results

### 2.1. Genomic Sequences

The assemblies for the trypanosomatid hosts of the strains TCC2435 and PNG-M02 consisted of 7753 (N50 = 22.5 kb) and 1740 contigs (N50 = 133.9 kb), with total lengths of 21.2 Mb and 23.7 Mb being similar to those of *Angomonas* spp. genomes (21–24 Mb) sequenced previously [16,17].

The genome assembly for the endosymbiont of *Angomonas ambiguus* TCC2435 (hereafter referred to as TCC2435 symbiont) contained 14 contigs with the total size of 803,474 bp and N50 of 126 kb. However, the contigs 8 and 12, comprising the ribosomal operon (~5.6 kb) and the EF-Tu gene (~1.2 kb), respectively, displayed a significantly higher coverage (Table S1) suggesting that they were present in more than one copy. Given that in the genomes of *Ca*. Kinetoplastibacterium spp. the first sequence invariantly has 3 copies and the second one has 2 (except for the very divergent *Ca*. K. sorsogonicusi), we estimate that the actual genome size should be bigger by at least 12.4 kb, i.e., ~816 kb. A similar genome length was obtained for *Ca*. K. crithidii from *A. ambiguus* PNG-M02 (hereafter referred to as PNG-M02 symbiont), the assembly of which contained a single scaffold of 816,901 bp. These values are smaller than that for the genome of the endosymbiont of *A. deanei* TCC036E (821,930 bp; hereafter referred to as TCC036E symbiont) used here as a reference, but are within the known size range for the genomes of bacteria from *Angomonas* spp. and *Strigomonas* spp. (810–830 kb) [16,17].

**Figure 1:**
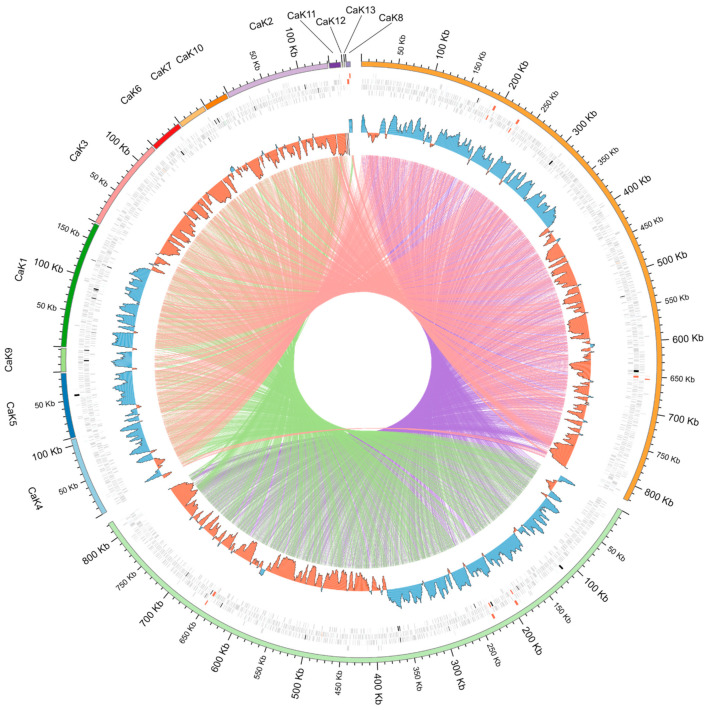
Comparison of the genomes of three *Ca*. K. crithidii strains. The rings in the outside-in direction mean: (i) genomic coordinates of scaffolds; (ii) predicted genes (protein-coding in grey, rRNA in red, tRNA in blue, tmRNA in orange, ncRNA in green, and pseudogenes in black); (iii) GC skew plot (negative values in red and positive ones in blue). The lines in the central area connect orthologous genes between the genomes in a pairwise manner.

The GC content of the genomes of the PNG-M02 and ATCC2435 symbionts was 30.33% and 30.65%, respectively. These values are very close to those for the genomes of *Ca*. K. crithidii ATCC036E (30.96%) and the symbionts from *Strigomonas* spp. (31.23–32.55%) [16]. Similarly to other *Ca*. Kinetoplastibacterium spp. [16,18] and bacterial endosymbionts in general [19,20], *Ca*. K. crithidii from *A. deanei* and the two *A. ambiguus* strains showed a very high level of gene order conservation with no detectable rearrangements (Figure 1 and Figure S1).

The overall genome sequence identity in the TCC2435/TCC036E, TCC2435/PNG-M02, and PNG-M02/TCC036E pairs was 90.8%, 90.4%, and 90.3%, respectively. These values are much higher than the interspecific similarity between the genomes of *Strigomonas* spp. symbionts (83–85%) or *Ca*. K. crithidii and *Ca*. K. desouzaii (73%) [16]. In agreement with the smaller size, the two bacterial genomes studied here were predicted to code for slightly smaller numbers of proteins: 729 and 726 for TCC2435 and PNG-M02 symbionts, respectively, as compared to 733 for the TCC036E symbiont (Table S2). However, the number of annotated pseudogenes in the two newly sequenced genomes was higher, with most of such sequences being frameshifted (Table S2). The distribution of the pseudogenes did not show any hotspots (Figure 1). Only 39 tRNA genes were predicted in the TCC2435 symbiont genome (which may be due to assembly fragmentation), whereas the genomes of PNG-M02 and TCC036E symbionts featured 43 and 44 such genes, respectively. The inspection of the tRNA lists for the three genomes revealed that they all differed from each other, but the differences consisted only in the number of redundant tRNAs, i.e., those with the same anticodon (Table S3).

### 2.2. Analysis of Orthologous Groups (OGs) of Proteins

**Figure 2:**
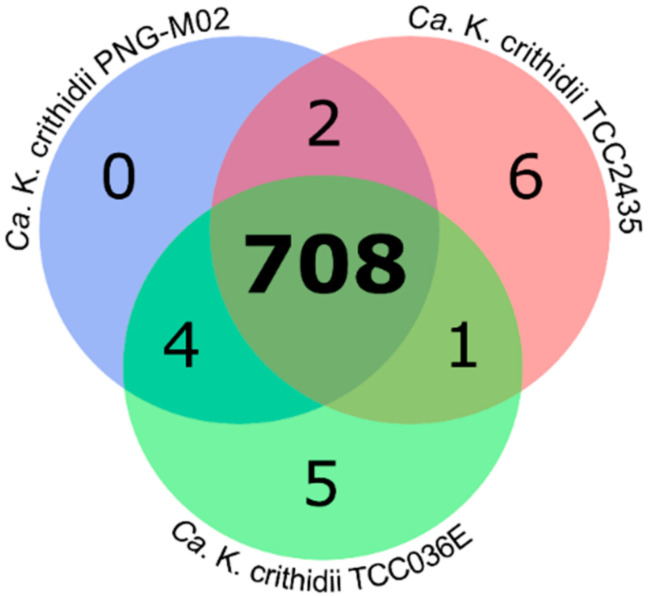
Sharing of orthologous groups of proteins encoded in the genomes of the three *Ca*. K. crithidii strains.

Only minor differences in gene content were revealed between the three analyzed endosymbiont genomes (Figure 2). The number of OGs present or absent only in one of the three genomes negatively correlated with the assembly quality, suggesting that at least some of the differences may be artifactual. Thus, the genome of the PNG-M02 symbiont assembled to a single contig based on PacBio and Illumina reads displayed the lowest numbers, whereas those for the fragmented assembly of the TCC2435 symbiont were the highest (Figure 2).

A detailed inspection of the “unique” genes revealed that most of them either represent pseudogenes with a degraded sequence, which leads to clustering them into separate OGs, or potentially spurious short ORFs with no BLAST hits in NCBI nr database (Table S4). After exclusion of annotated or suspected pseudogenes and sequences with no BLAST hits, only two “unique” genes remained, both in the TCC036E symbiont genome: a helix-turn-helix domain-containing protein and tetraacyldisaccharide 4′-kinase. Each of these two genes is present (but not invariably) in other *Ca*. Kinetoplastibacterium spp., suggesting their dispensability. The first one, appearing to be a transcription factor (based on blast results), is absent from the genomes of endosymbionts of all *Strigomonas* spp. The second gene codes for an enzyme phosphorylating a precursor of lipopolysaccharide (component of the outer membrane) and is absent from the genomes of *Ca*. K. galatii and *Ca*. K. oncopelti. This agrees with the previous observation that the functional category “cell wall, membrane, and envelope biogenesis” is overrepresented among lost and pseudogenized genes in the genomes of Strigomonadinae symbionts [16]. Similar results were obtained after the inspection of the OGs missing from one of the three genomes: most of them were associated with the synthesis of the cell wall or lipopolysaccharide (Table S4). In addition, the ribosome-associated translation inhibitor RaiA (also absent from the genomes of endosymbionts of all *Strigomonas* spp.) was not detected in the TCC036E symbiont genome, and a short hypothetical protein was absent from the genome of TCC2435 symbiont, although a potential homolog could be detected with an increased e-value threshold (Table S4).

### 2.3. Phylogenetic Analyses

**Figure 3:**
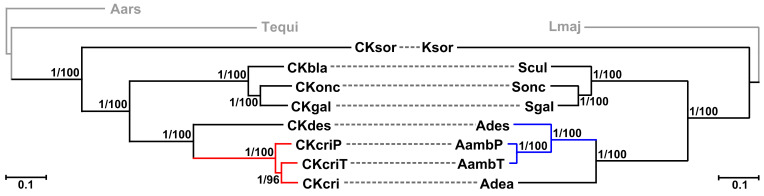
Juxtaposed maximum-likelihood phylogenomic trees of endosymbiotic bacteria and their respective trypanosomatid hosts. Outgroups are shown in grey. Dashed lines connect endosymbionts with their hosts, while colored branches point to the discrepancy between their phylogenies. Numbers at branches indicate bootstrap support and Bayesian posterior probability values, respectively. Scale bars show the number of substitutions per site. Organism codes: Lmaj, *Leishmania major*; Ksor, *Kentomonas sorsogonicus*; Scul, *Strigomonas culicis*; Sonc, *S. oncopelti*; Sgal, *S. galati*; Ades, *Angomonas desouzai*; AambP and AambT, *A. ambiguus* strains PNG-M02 and TCC2535, respectively; Adea, *A. deanei*; Aars, *Achromobacter arsenitoxydans*; Tequi, *Taylorella equigenitalis*; CKsor, *Candidatus* Kinetoplastibacterium sorsogonicusi; CKbla, *Ca*. K. blastocrithidii; CKonc, *Ca*. K. oncopeltii; CKgal, *Ca*. K. galatii; CKdes, *Ca*. K. desouzaii; CKcri, CKcriP, and CKcriT, *Ca*. K crithidii TCC036E, PNG-M02, and TCC2435, respectively.

For each of the two phylogenomic datasets used (431 and 1549 single-copy genes for bacteria and trypanosomatids, respectively), maximum-likelihood and Bayesian trees showed identical topology with all branches or all but one bearing maximal statistical supports (Figure 3). In accordance with the previous inferences, *Kentomonas sorsogonicus* represents here the earliest branch within the subfamily Strigomonadinae [13], whereas its bacterium, *Ca*. K. sorsogonicusi, occupies the same position among the endosymbionts of this trypanosomatid subfamily [18]. The relationships within the genus *Strigomonas* and their respective endosymbionts are also correlated, suggesting cospeciation of these two groups of organisms. The situation is different for the third genus of Strigomonadinae: although *Angomonas ambiguus* and *A. desouzai* represent sister taxa, the bacteria hosted by the former species are paraphyletic in respect to that of *A. deanei* (Figure 3). This suggests a single horizontal endosymbiont transfer from *A. ambiguus* to *A. deanei*, in contrast to the previous proposal that the transfer had the opposite direction [15]. The alternative explanation of this figure implies two independent endosymbiont switches from *A. deanei* to *A. ambiguus* and is less parsimonious.

**Figure 4:**
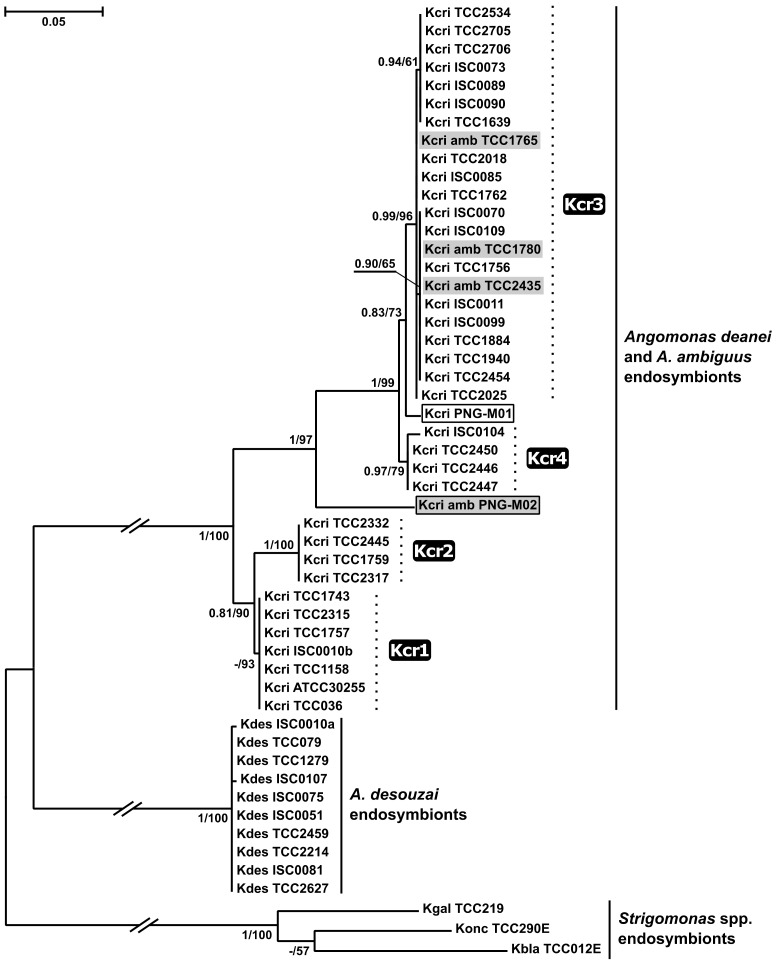
GAPDH-based maximum-likelihood phylogenetic tree of *Ca*. Kinetoplastibacterium spp. The endosymbionts of *Angomonas ambiguus* are highlighted in grey, the isolates from Papua New Guinea are boxed. The labels in black rectangles indicate individual subclades of *Ca*. Kinetoplastibacterium crithidii. Numbers at branches indicate bootstrap supports and Bayesian posterior probabilities, respectively. Scale bar show the number of substitutions per site. The tree is rooted with the sequences of *Strigomonas* spp. endosymbionts.

In order to clarify the situation, we performed an additional phylogenetic analysis using GAPDH gene sequences of *Ca*. Kinetoplastibacterium spp. This allowed investigating the relationships of these bacteria on a much larger set of strains, available from a previous study [15]. The phylogenetic trees inferred using maximum likelihood and Bayesian approaches displayed almost identical topologies differing only in the presence of a single very short branch with a very short length (Figure 4). They were congruent with the previously published GAPDH tree [15] and confirmed the unity of the symbionts from *A. deanei* and *A. ambiguus*, representing the same four subclades (Kcr1–Kcr4). As in the previous inference, all sequences of the endosymbionts from *A. ambiguus* from Brazil (isolates TCC1765, TCC1780, and TCC2435) nested within the Kcr3 subclade and displayed 100% identity to some sequences of the endosymbionts from *A. deanei* originating from the same country. However, *Ca*. K. crithidii from the Papuan *A. ambiguus* isolate PNG-M02 represented a separate lineage, sister to the KCr3+Kcr4 group. The identity of its GAPDH sequence to those of other *Ca*. K. crithidii was only ~91%, almost the same as the observed minimum within this bacterial species (90.8%). Interestingly, the endosymbiont of *A. deanei* PNG-M01, obtained from the same host species and the same locality as PNG-M02, was not related to the latter and nested within the KCr3 + Kcr4 group (Figure 4).

## 3. Discussion

Mutualistic endosymbioses of prokaryotes with eukaryotes are quite diverse in terms of involved taxa, time of origin, and level of interdependence, with the latter two factors usually being correlated: evolutionary older relationships demonstrate a higher level of integration [21]. In insects, whose relationships with prokaryotes have been studied quite intensively, symbionts permanently residing in the cytoplasm of the host cells usually display perfect co-evolutionary patterns in contrast to bacteria that do not have such a restriction and, therefore, can switch hosts and/or be replaced by other species [22]. In agreement with this trend, *Ca*. Kinetoplastibacterium spp. also show cospeciation with their trypanosomatid hosts and the only exception concerns the *A. deanei*–*A. ambiguus* pair, which shares a single endosymbiotic bacterium, *Ca*. K. crithidii. This was first detected using 16S rRNA gene sequences [7] and later confirmed by the analysis of bacterial GAPDH gene sequences [15].

Although being a rare phenomenon, the replacement of permanent endosymbionts is well known in insects [22] and presumably also occurs in ciliates [23,24]. The new bacterium in such a case originates from either a free-living or a facultatively symbiotic species and restores deteriorated functions of the old endosymbiont, whose genome degraded due to Muller’s ratchet [25]. The situation with the bacteria of *Angomonas* spp. is drastically different: both of them represent equally ancient endosymbionts and the replacement is combined with horizontal transfer between two related host species. This may appear unprecedented, but only when considering typical bacterial endosymbionts. A remarkable analogy can be found in mitochondria and plastids, the two kinds of organelles with prokaryote ancestry. The organellar capture (replacement of a mitochondrion or plastid of one species by that of another) also known as mitochondrial/plastid introgression (when it refers to genomes) has been described in a wide range of animals and plants and is usually associated with the formation of a hybrid zone between two species [26,27]. These species often have significantly different abundance levels resulting in asymmetrical introgression due to the contrast effects of genetic drift on small and large populations [28]. In general, introgression is driven by the prevalence of interspecific gene flow over the intraspecific one. In the case of mitochondria, this condition is met when dispersal is exerted predominantly by males (in some animals) or pollen (in conifers), not contributing the organelle to the progeny (due to maternal inheritance) and, thus, the intraspecific organellar gene flow for the colonizing species is close to zero [29,30].

Since the outcome of the interspecific interaction between *A. deanei* and *A. ambiguus* is similar to organellar capture, henceforth we will refer to it as endosymbiont capture. In order to understand the mechanism of this phenomenon, we summarize here the available data.

Out of the three *Angomonas* spp. described to date, *A. deanei* has the highest prevalence and the widest (potentially cosmopolitan) distribution. It was documented in various countries of Africa and South America, as well as in Papua New Guinea, Turkey, Czechia, and Russia [15,31,32]. Meanwhile, South America is currently the only known area for *A. desouzai*, whereas *A. ambiguus*, the rarest of the three species, has been also reported from Africa and Papua New Guinea [15,31,32]. All three species occur mostly in blowflies (Calliphoridae), although two clades of *A. deanei* apparently prefer Muscidae [15]. While it is unclear whether the single records of *A. deanei* and *A. desouzai* from Syrphidae [7] represent nonspecific infections, the first isolate of *A. deanei* from the predatory bug *Zelus leucogrammus* [33] undoubtedly is such a case [34].

Here, we sequenced and analyzed the genomes of *Ca*. K. crithidii from two *A. ambiguus* strains and compared them with the previously published genome of the endosymbiont from *A. deanei* TCC036E [16]. The three genomes display very similar sizes and GC content, a high level of nucleotide sequence identity and no significant differences in gene content. Based on these features, the three bacterial endosymbionts can be considered as members of a single species. Previously, the discussion of the discordance in the phylogenies of endosymbionts and their trypanosomatid hosts was based only on data concerning Brazilian strains, whereas here we also included those from a geographically distant area—Papua New Guinea. Our phylogenomic analysis confirmed the unity of the symbionts from *A. deanei* and *A. ambiguus*, but, due to the small number of included isolates, its results were inconclusive regarding the direction of the endosymbiont transfer. However, the phylogenetic analysis based on the bacterial GAPDH gene sequences allowed taking advantage of a larger *Ca.* K. crithidii sampling. It not only confirmed that the endosymbiont of *A. deanei* was captured by *A. ambiguus* but also demonstrated that this occurred more than once.

With little doubt, the occurrence of *Angomonas* spp. in the same blowfly hosts and the relatedness of the trypanosomatids are the factors that facilitate endosymbiont capture. It was demonstrated that *A. deanei* colonizes the host rectum and forms massive aggregates in the area of rectal papillae [32]. Presumably, upon mixed infections, cells of two different species may come into a close contact and attempt to undergo sexual process. In contrast to multicellular organisms, its successful completion is not required to create a new heritable nucleus-symbiont combination. Of note, a sex-independent (grafting-based) mechanism of chloroplast capture has been proposed for plants [35].

By analogy to organelle capture, the reported very low prevalence of *A. ambiguus* [15] explains the phenomenon to be observed as a unidirectional process with this species being an acceptor. The reason why only *A. deanei* but not *A. desouzai*, being more closely related to *A. ambiguus*, is observed as a donor may be also related to their relative abundance. However, we cannot exclude that this is just due to the small number of *A. ambiguus* strains analyzed to date. Importantly, the endosymbiont capture is a repeated process (there were at least two independent cases) and its incidence may depend on the local demographic situation. The identical GAPDH sequences of *Ca*. K. crithidii of *A. ambiguus* and several *A. deanei* strains from South America indicate a recent event in agreement with the data on the current drastically different prevalence of these two species in that area. Meanwhile, the sequences of this gene in the endosymbionts of the Papuan isolates of both trypanosomatid species obtained from the same population of blowflies were significantly different and positioned distantly on the phylogenetic tree. This might be a result of a relatively ancient endosymbiont capture. Regrettably, for the moment other isolates of these two species from Papua New Guinea and data on their prevalence in that region are not available.

In sum, the replacement of endosymbionts of *Angomonas ambiguus* by those of *Angomonas deanei* is a repeated process analogous to organelle capture described in multicellular organisms and apparently shares with the latter one of the underlying mechanisms.

## 4. Materials and Methods

### 4.1. Trypanosomatid Strains: Origin and Cultivation

In this work, two axenically cultivated strains of *Angomonas ambiguus* were used: (i) PNG-M02 from the blowfly *Chrysomya megacephala* collected in Nagada, Papua New Guinea [31]; and (ii) TCC2435 representing a clonal culture of TCC1780 isolated from *C. albiceps* in Campo Grande, Brazil [7]. The cultures were maintained at 27 °C in RPMI 1640 cultivation medium at pH 7.0 supplemented with 10% (*v*/*v*) fetal calf serum, 10 μg/mL of hemin, 100 units/mL of penicillin, and 100 µg/mL of streptomycin. In addition to cultures, DNA of the non-cultivated *A. deanei* strain PNG-M01 (from the same host species and location as PNG-M02) available from an earlier study [31] was used for PCR amplification of the bacterial GAPDH gene.

### 4.2. Genome Sequencing, Assembly, and Annotation

DNA extraction from both strains of *A. ambiguus* was performed by the classical phenol-chloroform method, without preceding separation of the endosymbiont and trypanosomatid cells. Sequencing of TCC2435 DNA was performed using Roche 454 GS-FLX Titanium (1.37 mln single-ended reads, 550 Mbp), and Illumina MiSeq (13 mln 2 × 250 bp paired-end reads) platforms. DNA of PNG-M02 strain was sequenced at Wellcome Sanger Institute using Illumina MiSeq and HiSeq 2500 technologies (2,4 mln 2 × 250 bp and 14.7 mln 2 × 125 paired-end reads, respectively) as well as PacBio RS II sequencing system (13,977 long reads, 321 Mbp). The corresponding raw reads are available from GenBank under the following accession numbers: ERS4809514 (PNG-M02) and SRR14208463, SRR14216068, SRR14216074, and SRR14209298 (TCC2435).

After processing the raw reads generated by Illumina and 454 platforms with Trimmomatic V. 0.39 [36] and those from the PacBio system with SMRT Analysis Suite (Pacific BioSciences, Menlo Park, CA, USA), the data quality was assessed using the FastQC v. 0.11.9 software (http://www.bioinformatics.babraham.ac.uk/projects/fastqc, accessed on 30 April 2020). Since the BLAST search against available genomic sequences of trypanosomatids revealed that the PNG-M02 sample was contaminated with DNA from *Crithidia fasciculata*, the data were filtered by mapping the preprocessed reads to the *C. fasciculata* genome Cf-C1 (TritrypDB v. 40) using BBmap v. 38.84 with the settings recommended for contaminant reads removal (http://sourceforge.net/projects/bbmap/, accessed on 6 May 2020). The genomic assembly for the PNG-M02 strain was made with hybridSPAdes v. 3.14.1 [37] using both Illumina and error corrected PacBio reads. The endosymbiont genome was identified using blastn and the closest known endosymbiont genome *Ca*. K. crithidii TCC036E. Two different assemblies were made for the strain TCC2435 with Newbler v. 2.7: (i) trypanosomatid-focused using only Illumina data (with “-large” option); and (ii) endosymbiont-focused using both 454 and Illumina reads. Genes were predicted with Companion [38] and Glimmer v. 3 [39] for the bacteria and their hosts, respectively. Gene annotation for the endosymbionts was performed with PROKKA v. 1.14.5 [40]. The assembled genome sequences have been deposited in GenBank under the Bioproject accession PRJNA673871.

### 4.3. Synteny Analysis of Bacterial Genomes

The single-scaffold genomic sequences of *Ca*. K. crithidii TCC036E (GCA_000340825.1) and *Ca*. K. crithidii PNG-M02 were circularized using Circlator v. 1.5.5 [41] and the dnaA gene was selected as a start in their linear representation. The scaffolds of *Ca*. K. crithidii TCC2435 genome were reordered and inverted to match the two abovementioned ones, following tripartite genome alignment and synteny analysis using Mauve v. 2015-02-13 [42]. Visualization of genomic alignment was prepared with Circos v. 0.69-9 [43].

### 4.4. Phylogenomic Analyses

Analyses of protein OGs were performed with OrthoFinder v. 2.3.11 [44] with the default settings. In addition to the sequences obtained in this work, the bacterial dataset (BD) comprised the previously published genomes of all six *Ca*. Kinetoplastibacterium spp. as well as those of *Achromobacter arsenitoxydans* and *Taylorella equigenitalis*, which were used here as outgroups (Table S5). The trypanosomatid dataset (TD) included previously published genomic sequences for Strigomonadinae and *L. major* Friedlin, which served as an outgroup (Table S5), as well as the generated earlier draft sequence of *Kentomonas sorsogonicus* [18]. Out of the total 1645 (BD) and 17,990 (TD) inferred protein OGs, 431 and 1549, respectively, included one protein per species and were used for the subsequent phylogenomic analyses. The amino acid sequences were aligned using Muscle v. 3.8.31 [45], trimmed with Gblocks v. 0.91b [46] and concatenated with FASconCAT-G v.1.04 [47]. The resulting supermatrices contained 133,474 (BD) and 658,788 (ED) positions with respective gap proportions of 0.4% and 5%. Maximum likelihood analyses were performed in RAxML v.8.2.11 [48] with automated selection of the substitution schemes for the partitioned model, linked edge lengths, and 100 bootstrap pseudoreplicates for branch support estimation. Bayesian inference was performed in MrBayes v. 3.2.6 [49] with “mixed” prior for amino acid substitution matrix and rate heterogeneity modelled using 4 discrete Γ-categories. Relative rates, substitution models, and Γ-distribution shape were unlinked across partitions. The analysis was run for 1,000,000 generations with every 100th tree sampled, and other parameters set by default.

### 4.5. Amplification and Phylogenetic Analysis of GAPDH Gene

The bacterial GAPDH gene of the strain PNG-M01 was amplified and sequenced using the newly designed primers KAGF1 (5′-ATTTTAAGAGCTCATTACGAAGGT-3′) and KAGR1 (5′-GATCTTGCCCTACGCAAATC-3′). The obtained sequence was deposited in GenBank under the accession number MW161049. Other sequences of this gene from the endosymbionts of *Angomonas* and *Strigomonas* available in the GenBank were collected (Table S6) and aligned with MAFFT v. 7.471 using L-INS-I algorithm [50]. Maximum Likelihood analysis was accomplished in IQ-TREE v. 2.0.5 [51] with the best substitution model (TIM2 + F + I) selected by the built-in ModelFinder [52]. The statistical support of branches was estimated by the standard bootstrap method with 1000 pseudoreplicates. Bayesian inference was accomplished in MrBayes v. 3.2.7 under the GTR + I model with run parameters as described above.

## Data Availability

The data used in this study are publicly available from GenBank under the following accession numbers: ERS4809514 (PNG-M02 raw reads), SRR14208463, SRR14216068, SRR14216074, and SRR14209298 (TCC2435 raw reads), Bioproject PRJNA673871 (generated assemblies).

## Supplementary Materials

The following are available online at https://www.mdpi.com/article/10.3390/pathogens10060702/s1, Figure S1: MAUVE alignment of three genomes of *Ca*. Kinetoplastibacterium spp., Table S1: Read coverage of the genomic contigs of the TCC2435 symbiont, Table S2: Summary statistics on the genes encoded in the three *Ca*. K. crithidii genomes, Table S3: Comparison of lists of tRNA genes in the genomes of endosymbionts from *Angomonas* spp., Table S4: Presence and absence of OGs in the genomes of *Ca*. K. crithidii, Table S5: Previously published genomic sequences used in the phylogenomic analyses, Table S6: GAPDH gene sequences from *Ca*. Kinetoplastibacterium sequences retrieved from GenBank for phylogenetic analysis.
