# Supplementary figures and images for: Endosymbiont Capture, a Repeated Process of Endosymbiont Transfer with Replacement in Trypanosomatids Angomonas spp
Source: Pathogens. 2021 Jun 4;10(6):702. doi: 10.3390/pathogens10060702 (PMC8229890; doi:10.3390/pathogens10060702)

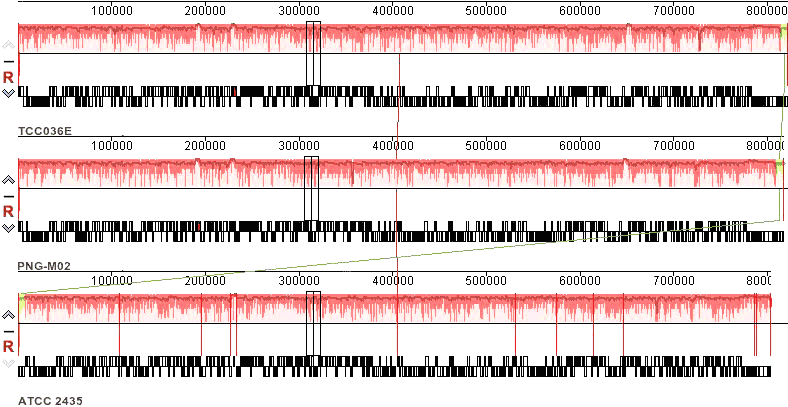

Supplement: Supplementary file 1 [file pathogens-10-00702-s001.zip › FigS1.png]
